# Silver Nanoparticles-Composing Alginate/Gelatine Hydrogel Improves Wound Healing In Vivo

**DOI:** 10.3390/nano10020390

**Published:** 2020-02-23

**Authors:** Flavia Resende Diniz, Romerito Cesar A. P. Maia, Lucas Rannier M. de Andrade, Luciana Nalone Andrade, Marco Vinicius Chaud, Classius Ferreira da Silva, Cristiane Bani Corrêa, Ricardo Luiz C. de Albuquerque Junior, Luiz Pereira da Costa, Su Ryon Shin, Shabir Hassan, Elena Sanchez-Lopez, Eliana Barbosa Souto, Patricia Severino

**Affiliations:** 1 Tiradentes University (UNIT) and Institute of Technology and Research (ITP), Av. Murilo Dantas 300, Aracaju 49032-490, Brazil; flavinhadiniz_se@hotmail.com (F.R.D.); romeritocesar@hotmail.com (R.C.A.P.M.); rannier.andrade@outlook.com (L.R.M.d.A.); luciana.nalone@hotmail.com (L.N.A.); ricardo_albuquerque@unit.br (R.L.C.d.A.J.); lupeco7@hotmail.com (L.P.d.C.); 2 Department of Pharmaceutical Technology, Faculty of Pharmacy, University of Coimbra, Pólo das Ciências da Saúde, Azinhaga de Santa Comba, 3000-548 Coimbra, Portugal; 3 Department of Technological and Environmental Processes, Sorocaba University (UNISO), Rod. Raposo Tavares, Km 92.5, Sorocaba 18023-000, Brazil; marco.chaud@prof.uniso.br; 4 Department of Chemical Engineering, Federal University of São Paulo (UNIFESP), Rua Prof. Artur Riedel, 275, Diadema 09972-270, Brazil; classiusferreira@yahoo.com.br; 5 Department of Morphology, Federal University of Sergipe (UFS), Avenida Marechal Rondon, São Cristovão 49100-000, Brazil; crisbani@gmail.com; 6 Center for Biomedical Engineering, Department of Medicine, Brigham and Women’s Hospital, Harvard Medical School, 65 Landsdowne Street, Cambridge, MA 02139, USA; sshin4@bwh.harvard.edu (S.R.S.); SHASSAN@bwh.harvard.edu (S.H.); 7 Department of Pharmacy, Pharmaceutical Technology and Physical Chemistry, Faculty of Pharmacy and Food Sciences, Av. Joan XXIII 27-31, 08028 Barcelona, Spain; esanchezlopez@ub.edu; 8 Institute of Nanoscience and Nanotechnology (IN2UB), University of Barcelona, Av. Joan XXIII 27-31, 08028 Barcelona, Spain; 9 Biomedical Research Networking Centre in Neurodegenerative Diseases (CIBERNED), 28031 Madrid, Spain; 10 CEB—Centre of Biological Engineering, University of Minho, Campus de Gualtar, 4710-057 Braga, Portugal; 11 Tiradentes Institute, 150 Mt Vernon St, Dorchester, MA 02125, USA

**Keywords:** sodium alginate, gelatin, silver nanoparticles, antimicrobial activity, healing

## Abstract

Polymer hydrogels have been suggested as dressing materials for the treatment of cutaneous wounds and tissue revitalization. In this work, we report the development of a hydrogel composed of natural polymers (sodium alginate and gelatin) and silver nanoparticles (AgNPs) with recognized antimicrobial activity for healing cutaneous lesions. For the development of the hydrogel, different ratios of sodium alginate and gelatin have been tested, while different concentrations of AgNO_3_ precursor (1.0, 2.0, and 4.0 mM) were assayed for the production of AgNPs. The obtained AgNPs exhibited a characteristic peak between 430–450 nm in the ultraviolet-visible (UV–Vis) spectrum suggesting a spheroidal form, which was confirmed by Transmission Electron Microscopy (TEM). Fourier Transform Infra-red (FT–IR) analysis suggested the formation of strong intermolecular interactions as hydrogen bonds and electrostatic attractions between polymers, showing bands at 2920, 2852, 1500, and 1640 cm^−1^. Significant bactericidal activity was observed for the hydrogel, with a Minimum Inhibitory Concentration (MIC) of 0.50 µg/mL against *Pseudomonas aeruginosa* and 53.0 µg/mL against *Staphylococcus aureu*s. AgNPs were shown to be non-cytotoxic against fibroblast cells. The in vivo studies in female Wister rats confirmed the capacity of the AgNP-loaded hydrogels to reduce the wound size compared to uncoated injuries promoting histological changes in the healing tissue over the time course of wound healing, as in earlier development and maturation of granulation tissue. The developed hydrogel with AgNPs has healing potential for clinical applications.

## 1. Introduction

In 1962, G.D. Winter pioneered the development of occlusive dressings with healing agents for skin regeneration [1,2]. For wound healing, the dressing should be able to keep the temperature constant, favor the healing process, protect new cells, and exhibit antimicrobial activity [3,4,5]. 

Biopolymers, such as sodium alginate and gelatin, are interesting materials for wound healing because they are biocompatible, biodegradable, and bioabsorbable [6]. Sodium alginate is a natural polymer derived from brown algae *Phaeophyta*, formed by monomers of residues of L-guluronic acid and D-mannuronic present in the cell wall and intercellular space [7]. Gelatin is a protein derived from the denaturation of collagen, obtained from the skin and bones of animals. Collagen denaturation occurs due to the breakdown of hydrogen interactions, and the triple helices are separated from each other assuming a random structure. It exhibits relevant characteristics such as plasticity, adhesiveness, cell adhesion capacity, and the possibility for tissue growth. The association of these two biopolymers has been studied due to their unique characteristics, namely, low toxicity, elasticity and capacity for reabsorption of constituent materials [8]. The ionic interaction between sodium alginate and gelatin occurs due to the presence of ionizable amino and carboxyl groups, in addition to the hydrogen interactions between the amine and carboxyl group [9]. According to Peppas et al. (2000), hydrogels are the result of hydrophilic polymeric networks capable of absorbing large amounts of water or biological fluids [10]. In the case of gelatin, the crosslinking can be chemical in nature, formed by irreversible covalent bonds, or physical if formed by reversible covalent bonds. As an example, ionizing or non-ionizing radiation can be used to crosslink polymer chains and prepare a hydrogel.

Polymeric hydrogels containing silver nanoparticles (AgNPs) have attracted great attention for a set of applications in the biomedical field, attributed to the antimicrobial and healing properties of silver [11,12]. The antimicrobial activity of silver has been known for many years, and literature shows that in small concentrations, it is considered safe for human use. Studies suggest that the decrease of the particle size changes the electronic structure of AgNPs, favoring their antimicrobial activity. Such property is associated with the slow release and oxidation of Ag^+^ in the biological environment. Besides its capacity to cross the cellular membrane, Ag^+^ affects cell division, directly causing the death of the microorganism [13]. Various methodologies have been proposed for the production of AgNPs [14,15], while the most common approach is based on the stirring of one or more polymers with silver nitrate [16]. The choice of the polymer to stabilize AgNPs is based on its capacity to create voids within the polymeric network during the swollen phase, which will serve as the nucleation site and growth of the nanoparticles. For application onto the wound, the dressing may be applied in the form of a hydrogel, which can be produced from natural, synthetic, or semi-synthetic polymers. Those of natural origin are the most commonly used because of their biodegradability and biocompatibility with the human body [17]. Examples are alginate, chitosan, heparin, chondroitin, proteoglycans, collagen, gelatin, fibrin, keratin, and silk fibroin [18]. The use of alginate and gelatin in tissue engineering, drug delivery, and wound dressings [11,19,20,21,22,23,24] is very well documented in the literature. If formulated with AgNPs, a hydrophilic environment is required to facilitate the release of the nanoparticles from the polymeric network, maintaining the wound hydrated to promote better healing. The aim of this work has been the development and characterization of an AgNPs-containing alginate/gelatin nanocomposite to be used in the healing of cutaneous wounds.

## 2. Materials and Methods

### 2.1. Materials

Sodium alginate (Protonal^®^RF6650, FMC Corporation, Philadelphia, Pennsylvania, USA) was donated by FMC (Campinas, São Paulo, Brazil). Silver nitrate was purchased from Química Contemporânea (Indaiatuba, São Paulo, Brazil). Bovine gelatin was obtained from Gelnex (Itá, Santa Catarina, Brazil). Other chemicals of analytical grade were obtained from Sigma-Aldrich (Darmstadt, Germany). Water from Millipore Milli^®^-Q system (Merck KGaA, Darmstadt, Germany), home supplied, was used for the preparation of the aqueous solutions.

### 2.2. Production of AgNPs-Composing Alginate/Gelatin Hydrogel

The production of AgNPs-composing alginate/gelatin hydrogel was adapted from Rescignano et al. [25]. Briefly, sodium alginate and gelatin were firstly solubilized, separately, in water at the concentration of 2% (m/v) each, followed by the production of a solution of sodium alginate and gelatin in different proportions (i.e., 80:20, 50:50 or 20:80) by homogenization for 2 h at 500 rpm with a mechanical stirrer (Biomixer 78HW-1, Biomex Biotecnologia, Ribeirão Preto, São Paulo, Brazil), to obtain a hydrogel. AgNO_3_ was simultaneously dissolved in water in different concentrations (1.0, 2.0, and 4.0 mM) and slowly added to the hydrogel, under mechanical stirring for 2 h at 500 rpm to produce AgNPs.

### 2.3. UV–VIS Spectrophotometer Analysis

UV–VIS absorption spectra were recorded on a UV–Vis spectrophotometer (FEMTO 800Xi, Femto Indústria e Comércio de Instrumentos Ltda., São Paulo, Brazil) in a scan range of 300–700 nm. All samples were previously dispersed in water, and analysis was run at room temperature.

### 2.4. Fourier-Transform Infra-Red Analysis

Fourier Transform Infra-red (FT–IR) analysis was used to investigate the chemical interaction between components of the formulations (Agilent, Cary 630, Santa Clara, CA, USA) with 32 accumulations of 4000–500 cm^−1^, with resolution 2 cm^−1^ in potassium bromide (KBr) tablets.

### 2.5. Thermal Analysis

The thermal behavior was assessed by DSC (DSC Q20 TA Instruments, New Castle, DE, USA). The samples were previously weighted (3–5 mg) in an aluminum pan and sealed hermetically. Analyses were carried out under an inert atmosphere (45 mL/min of N_2_). DSC thermograms were scanned in the first heating run at a constant rate of 10 °C/min and a temperature range of 25–500 °C. TGA was conducted in a dynamic nitrogen atmosphere (50 mL/min) at a heating rate of 10 °C/min (Shimadzu DTG 60, Tokyo, Japan). The percentage of mass loss was recorded from the ambient temperature up to 800 °C.

### 2.6. Transmission Electron Microscopy (TEM)

Micrographs were obtained by TEM-MSC JEOL 2100 (JEOL Ltd., Tokyo, Japan) operating with 200 kV acceleration. Aqueous dispersions were dripped directly onto the copper grid, while the semi-solid hydrogels were previously dispersed in water, applying ultrasounds for 60 min and then dripped onto carbon-coated (carbon electrolyte 400 mesh Copper grids) (~4 nm). 

### 2.7. Biological Assays

#### 2.7.1. Viability Assays

The test was performed based on ISO 10993-5 [26]. The cytotoxicity assay was carried out against human fibroblast L929 cell lines [27,28]. Samples of AgNO_3_, pure gelatin, pure alginate, and alginate-gelatin with AgNPs were evaluated. Briefly, L929 cells were seeded in 96-well culture plates (2 × 10⁴ cells/well) and cultured in Dulbecco’s Modified Eagle Medium (DMEM) medium containing NaHCO_3_ (1.2 g/L,), ampicillin (0.025 g/L), streptomycin (0.1 g/L), supplemented with 10% fetal bovine serum (FBS). The negative control group was treated with the vehicle used to dilute the drug (DMSO 5%). For positive control, doxorubicin solution (100 μg/mL) was used [29]. Cell viability was assessed by the colorimetric method using Methyl-thiazolyl-tetrazolium (MTT). All reagents used in cell culture have been supplied by Sigma Chemical Co. (St. Louis, MO, USA). MTT solution (0.05%) was placed in contact with the cells, which were incubated at 37 °C for 3 h. After that, MTT was removed and added dimethyl sulfoxide (DMSO) for 10 min for solubilization of the tetrazolium salt crystals, and then the optical density (OD) reading was performed on an automated plate reader (ELISA) at a wavelength of 570 nm. The tests were conducted in quadruplicate and then normalized. The percentage of cell viability was calculated using the following equation, in which Abs stands for the absorbance of each respective solution:

(1) $$\%Cell\;viability=\frac{Abs\;\left(treated\;cells\right)-Abs\;\left(blank\right)}{Abs\;\left(negative\;control\right)-Abs\;\left(blank\right)}\times 100$$

#### 2.7.2. Minimum Inhibitory Concentration (MIC)

*Staphylococcus aureus* (ATCC 25923) and *Pseudomonas aeruginosa* (ATCC 27853) strains were used for MIC assay. Colonies were harvested and resuspended to 1.5 × 108 CFU/mL (turbidity equivalent to 0.5 McFarland standard scale). The bacterial solution was diluted to 1 × 105 CFU/mL in Mueller Hinton Broth with 100 µL of gelatin, sodium alginate, AgNO_3,_ and hydrogel (1, 2, and 4 mM). The negative control was 0.1 mL of Mueller Hinton Broth, and the positive control was 0.05 mL Mueller Hinton Broth and 0.05 mL bacterial solution at 1 × 10^5^ CFU/mL dilution. The microdilution method has been used [30]. Plates were incubated at 37 °C for 20 h.

#### 2.7.3. Wound Healing Test

Adult Females Wistar rats (250 ± 50 g) were used. The Animal Research Ethics Committee of the Tiradentes University approved the *in vivo* procedures set in the Protocol No. 011116R, in compliance with the Guide for the Care and Use of Laboratory Animals published by the US National Institutes of Health. The animals were divided into three groups (*n* = 9, total 27 animals) and housed under conditions of controlled temperature (22 ± 1 °C) with a light/dark cycle of 12/12 h with free access to food and water. It was anesthetized with intraperitoneal injection composed of 1 mL ketamine (50 mg/kg) and 1 mL xylazine (20 mg/kg). The dorsal region was trichotomized, sterilized with a solution of polyvinylpyrrolidone-iodine, and the wound was done with a punch with 8 mm of width. In the immediate postoperative period, the animals received 10 mg/kg of ketoprofen intramuscularly for three days as a prophylactic dose of postoperative symptomatology, and the formulation was applied in the wound. Groups and each group identified all animals according to the number of days for analysis of the cicatricial process (3rd, 7th, and 14th day) until euthanasia in a CO_2_ chamber. The groups were divided into Control Group (G_CTR_), Group hydrogel sodium alginate/gelatin (80:20) (G_H_), and Group hydrogel with AgNP 4 mM AgNO_3_ (G_HP_) [31]. In the postoperative period, the lesions were controlled with photographic images (Sony brand camera, 10.1 megapixels) and measured with a digital caliper at the inner edges of the diameter at zero, three, seven, and fourteen postoperative days. The images were processed with the ImageJ^®^ software. After the animals were sacrificed, the equivalent specimens were removed from the scar area with margins of 3.0 mm for histological characterization.

#### 2.7.4. Histomorphology Analysis

Removal of the specimens was equivalent to a scar area with a 0.5 cm margin of whole skin around the lesion, with depth up to the first muscle layer. The removed samples were fixed in 10% buffered formalin solution (pH 7.4) for 48 h. Subsequently, they were dehydrated in ethanol solutions at 70, 95, and 100 °GL (Gay-Lussac degrees), diaphanized in xylol to make the tissue transparent, and embedded in paraffin. Histological sections (5 µm thick) were obtained from paraffin-embedded samples and subsequently subjected to Hematoxylin/Eosin (HE) staining. All the histological analyses were carried out by two observers, blinded to the treatment. 

### 2.8. Statistical Analyses

The data collected from in vitro and in vivo experiments were expressed as the mean ± standard error of the mean (SEM), and the differences among experimental groups were evaluated using one-way analysis of variance ANOVA followed by the Tukey’s test. Values of p < 0.05 were considered statistically significant. All statistical analyses were carried using the GraphPad program (Intuitive Software for Science, San Diego, CA, USA).

## 3. Results and Discussion

Hydrogels of different sodium alginate/gelatin ratios (80:20, 50:50, and 20:80) have been produced and characterized. The ratio 80:20 exhibited higher consistency, suitable for topical application. The dressings should be flexible enough to allow adherence to the skin/tissue to be treated for a prolonged period, offering more comfort and convenience to the patient [32]. The formation of strong intermolecular interactions between sodium alginate and positively charged polymers is responsible for the increased consistency, including hydrogen bonds and electrostatic attractions that occur at higher concentrations of both polymers [7,28]. According to Li et al., the interaction between alginate and gelatin occurs due to the presence of amino and carboxyl ionizable groups, as well as hydrogen interactions formed by the functional carboxylic and hydroxyl group [9]. The use of sodium alginate and gelatin hydrogels, as these are simple, efficient, and reproducible pharmaceutical dosage forms, becomes feasible in the field of tissue repair.

After producing the hydrogels, silver nitrate was incorporated in the hydrogel in distinct concentrations (1.0, 2.0, and 4.0 mM), showing increasing changes in color, from white to dark brown. The dark brown indicates nanoparticle production. AgNPs have been synthesized applying a green approach and using a natural biopolymer. The polymers show the presence of hydroxyl and carboxylic acid groups in their polymeric units favoring the Ag+ chelate production by the adjacent –OH and –COOH groups of alginate and gelatin. The Ag+ also acts as a potent oxidizing agent for organic compounds [33]. 

**Figure 1:**
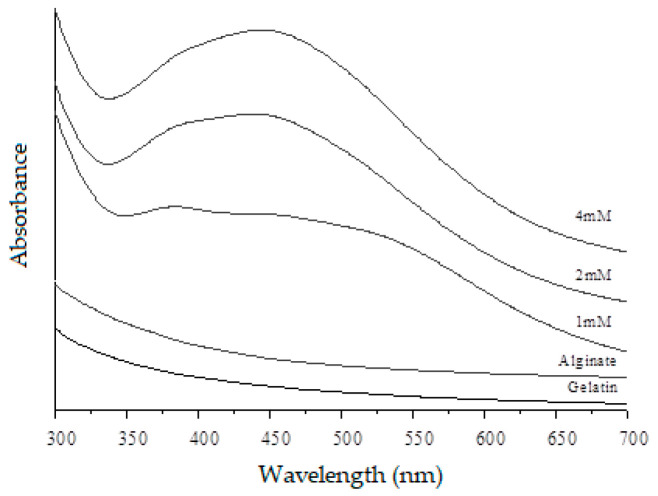
Ultraviolet-visible (UV–Vis) absorption spectra of sodium alginate, gelatin, and hydrogel with silver nanoparticles at different concentrations (1 mM, 2 mM, and 4 mM).

The light incident on the nanoparticles produces oscillations in the electrons that are on their surface, with the consequent absorption of electromagnetic radiation. The optical properties of the AgNPs-loaded hydrogels were evaluated by measuring the spectrum of UV–Vis spectroscopy (Figure 1). It identifies the evolution of the plasmon band as a function of the concentration of Ag^+^. In the analysis of a spectrum UV–Vis, the bands of the plasmons are characterized by the absorption of the AgNPs showing a maximum wavelength, which indicates the presence of AgNPs caused by the excitation of electromagnetic waves (plasmon) in the surface. The spheroidal AgNPs absorb at a wavelength between 390–440 nm, which may vary depending on the size and interaction between the particles. The nanoparticle absorption spectra of this analysis showed a maximum band around 430–450 nm suggesting a spheroidal form. Other researchers developed hydrogel with natural polymer incorporating AgNPs and obtained similar results [34,35].

**Figure 2:**
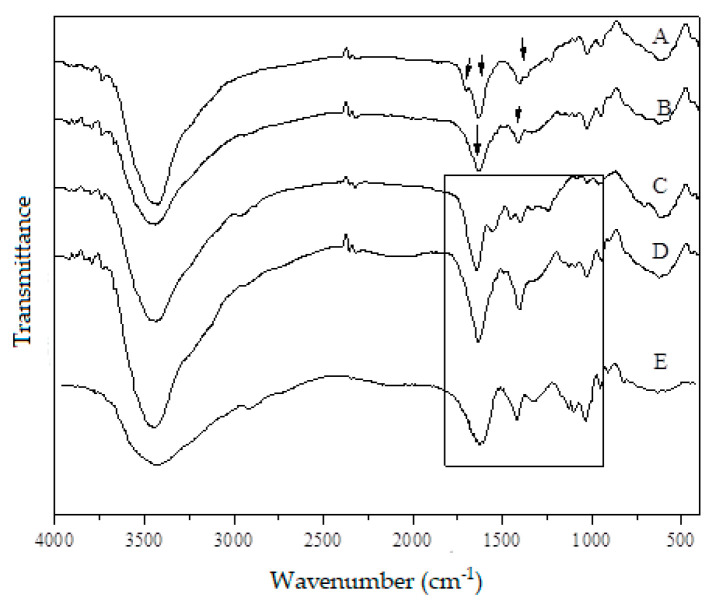
Fourier Transform Infra-red (FT–IR) spectra of (**A**) sodium alginate, (**B**) gelatin, and of hydrogels with silver nanoparticles at different concentrations (**C**) 1 mM, (**D**) 2 mM, and (**E**) 4 mM. Arrows of spectrum A correspond to the bands around 1650 cm^−1^ (C=C stretching), 1250 cm^−1^ (C–O–C stretching); arrows of spectrum B correspond to 1640 cm^−1^ and 1510 cm^−1^ of amide carbonyl (C=O and C=N stretching vibration); spectra of the hydrogel (square of C, D, and E) are the bands around 1600–1500 cm^−1^.

FT–IR was used to identify the characteristic bands of the present groups and to observe the possible interaction between the functional groups of the molecules composing the formulation. Figure 2 shows the FT–IR spectra of sodium alginate, gelatin, and hydrogel with silver nanoparticles (1 mM, 2 mM, and 4 mM). The characteristic absorption bands at 1602 cm^−1^ and 1424 cm^−1^ shown in the FT–IR spectrum of sodium alginate correspond to the amide carbonyl group [36]. Also, the bands around 1650 cm^−1^ (C=C stretching), 1250 cm^−1^ (C–O–C stretching), and 1250 and 1100 cm^−1^ (C–O stretching) are attributed to the polysaccharide structure (arrows of spectrum A, Figure 2). The spectrum of gelatin is characterized by 1640 cm^−1^ and 1510 cm^−1^ (arrows of spectrum B, Figure 2), corresponding to amide carbonyl (C=O and C=N stretching vibration). The bands from 1207 cm^−1^ to 1020 cm^−1^ are references of amino and alkyl chains. From the spectra of the hydrogel (square of spectra C, D, and E), the band around 1600–1500 cm^−1^ shifts toward higher wavenumbers, and the intensity of bands increase. Comparing the pure polymer with the blend of sodium alginate and gelatin (80:20), these changes suggest the formation of strong intermolecular interactions as hydrogen bonds and electrostatic attractions between polymers [37]. The bands around 2920, 2852, 1500, and 1640 cm^−1^ are typically found in hydrogels [38].

**Figure 3:**
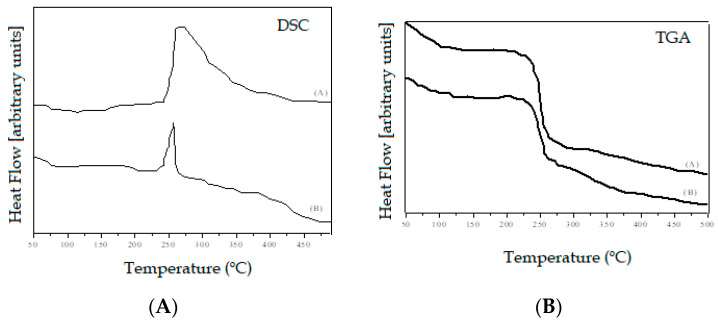
Differential Scanning Calorimetry (DSC) analysis (left-hand panel) of the (**A**) hydrogel alginate/gelatin (80:20) and (**B**) hydrogel with silver nanoparticles (AgNPs) (4 mM); Thermogravimetric (TGA) analysis (right-hand panel) of (**A**) hydrogel alginate/gelatin (80:20), and (**B**) hydrogel with AgNPs (4 mM).

The thermal behavior of the hydrogel was analyzed using DSC and TGA [39]. Samples of hydrogel sodium alginate/gelatin (80:20) and hydrogel with AgNP 4 mM AgNO_3_ are shown in Figure 3. The endothermic peaks at 230–250 °C (Figure 3a) were attributed to the fusion of the samples. However, a hydrogel with AgNPs showed peaks of lower intensity. Martins et al. [40] suggest that the AgNPs surface has a passive layer, which increases the charge density of hydrogel. This fact stabilizes the hydrogel, and the endothermic peak intensity decreased.

As shown in Figure 3b, for all tested samples, the mass loss occurred in two phases. The first phase was characterized by a discrete loss of initial mass that occurred at a temperature of 100 °C, due to the evaporation of water. The second mass decrease was identified at the temperature threshold between 230–260 °C, attributed to the thermal decomposition of the hydrogels. Upon reaching the maximum decomposition peak (260 °C), the samples lose approximately 50% of their initial mass.

Comparing with the literature [41], sodium alginate and gelatin showed less thermal stability than the produced hydrogel. The higher thermal stability of the hydrogel suggests that crosslinked provided thermal resistance and might be the lower release of small molecules like CO_2_. Sabadini et al. [42] reported similar thermogravimetric results in hydrogel analysis consisting of sodium alginate and chitosan. At a temperature close to 83 °C, the evaporation of water occurred, a chemical process facilitated by the high affinity of these polymers to the aqueous medium. In their study, the initial mass loss occurred at a temperature of 100 °C, which corroborates similar activities of the hydrogel, consisting of sodium alginate and gelatin. The peaks of degradation of the hydrogel mass occurred in temperatures between 239–248 °C, values close to those recorded in our study with the variance between 230–260 °C.

**Figure 4:**
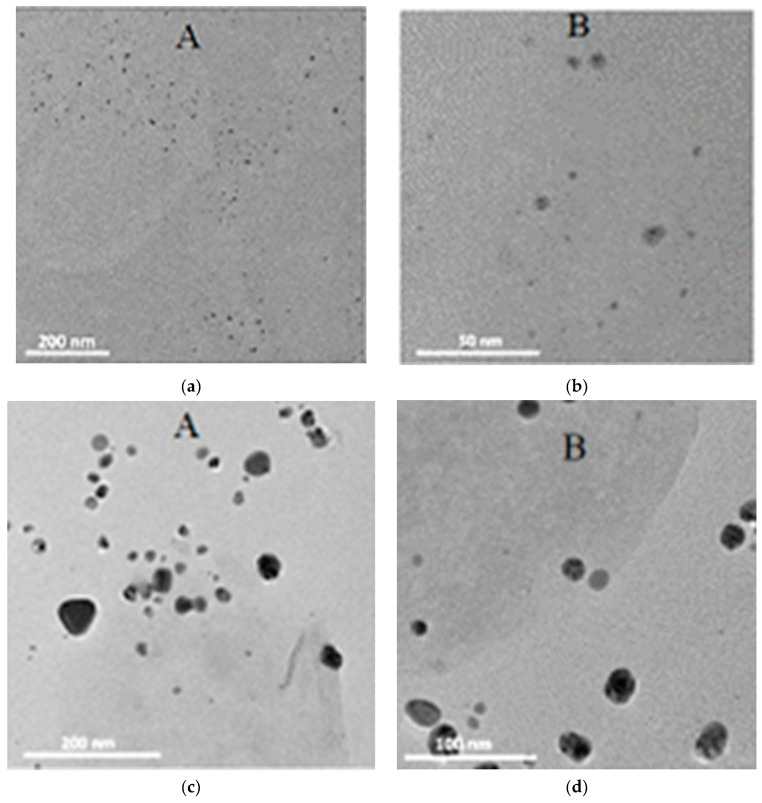
Electron micrographs of the hydrogels with silver nanoparticles at different concentrations: 1 mM on the 200 nm scale (**a**) and 50 nm scale (**b**); 4 mM on the 200 nm (**c**) and 100 nm scale (**d**).

TEM was performed to determine the morphology of the AgNPs (Figure 4), which exhibited a spherical shape and size dependent on the concentration of silver nitrate. Figure 4a,b shows the hydrogel with 1 mM AgNPs of approximately 7.5 to 8.3 nm. Figure 4c,d shows the hydrogel with 4 mM AgNPs of approximately 20 and 34 nm.

Sodium alginate and gelatin act directly as stabilizers, thereby avoiding aggregation of the particles. AgNPs, on the other hand, prevent degradation of the hydrogel either by exposure to light or by oxidation, avoiding possible chemical reactions. The spherical shape of AgNPs has also been reported in the literature [43]. At the nanoscale, most metals tend to agglomerate due to their high surface tension since the particle size results in a large surface area, and most of them have a size around 27 nm but vary in a parameter of 5–50 nm.

Biological evaluation of nanoparticles-based formulations is a way of verifying the potential toxicity arising from wastes formed during the production process. To be used as a biomaterial, the system must be biocompatible, i.e., it must interact with the physiological environment without undergoing changes or causing tissue damage (ISO E. 10993-5, 2009). ISO 10993-5 suggests that the in vitro cytotoxicity test is the first to evaluate the biocompatibility of a material and in this case, the toxic effects are assessed in normal cells.

**Figure 5:**
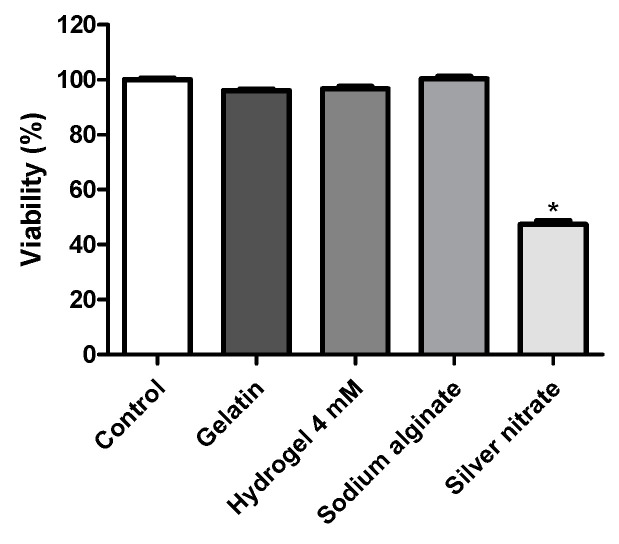
Cell viability assay of gelatin, hydrogel containing 4 mM of AgNPs, sodium alginate, and silver nitrate of human L929 fibroblasts, determined by the methyl-thiazolyl-tetrazolium (MTT) assay after 24 h of incubation. The vehicle used to dilute the drug (dimethyl sulfoxide, DMSO 5%) was used as the negative control (100% viability). The data correspond to the mean ± SEM of four independent experiments. * *p* < 0.05 compared to the control group using one-way analysis of variance followed by Tukey’s test.

A viability assay was carried out on human fibroblast L2929 testing all components used for the production of silver nanoparticles at the maximum concentration of 150 μg/mL in DMSO 5%. The results are shown in Figure 5. From all tested samples, only the silver nitrate induced the inhibition of cell proliferation, showing a statistically significant difference (p < 0.05) when compared to the negative control (DMSO). None of the remaining samples showed statistically significant differences when compared to the negative control (p > 0.05). The negative control group was treated with DMSO 5% only and presented 100% cell viability. Sodium alginate, gelatin, and 4 mM silver nanoparticles treated cells resulted in 100%, 96.66%, and 96% cell viability, respectively. On the other hand, pure silver nitrate induced 47.33% of living cells, thus demonstrating its cytotoxic potential on fibroblasts. The results indicate the safety and biocompatibility of the samples tested in this cell line. Any cytotoxic effect of these compounds may be due to their adhesion to the cell membrane, internalization and degradation of products in the cell culture medium or inside the cells.

The antimicrobial activity test was performed in vitro using Gram-positive (*Staphylococcus aureus*) and Gram-negative (*Pseudomonas aeruginosa*) bacteria. MIC tests performed with gelatin, sodium alginate, silver nitrate, and hydrogel (1, 2, and 4 mM) are shown in Table 1. Gelatin and sodium alginate alone (without the incorporation of AgNPs) did not exhibit antimicrobial activity; the bacterial growth was therefore expected. These natural polymers contribute both to achieve the adequate consistency of the hydrogel through hydrogen bonds interaction, formed between the functional carboxylic and hydroxyl group. All tested concentration ratios of AgNPs incorporated in the hydrogel showed bactericidal activity. The Minimum Inhibitory Concentration (MIC) values recorded for the treatment with Hydrogels at 1.0 mM and 2.0 mM remained constant for gram-negative and gram-positive bacteria. The increased hydrogel concentration up to 4.0 mM induced the reduction of the MIC values in both strains. A significant bactericidal action was observed both against *Pseudomonas aeruginosa* with a minimum bacterial increase of 0.50 µg/mL and against *Staphylococcus aureus* with a minimum bacterial increase of 53 µg/mL (Table 1). This result corroborates the study of Rescignano et al. [44] in which all hydrogels incorporating AgNPs showed inhibition of bacterial growth, suggesting that the antimicrobial activity is associated with the direct contact of the AgNPs with bacteria.

As AgNO_3_ is toxic to microorganisms, it already exhibits bactericidal action. Therefore, the use of AgNO_3_ was also evaluated in this study, obtaining an expected result with the absence of bacterial growth when tested against *Pseudomonas aeruginosa* and *Staphylococcus aureus*. Thus, the antimicrobial activity related to the use of AgNPs occurs in Gram-positive and Gram-negative bacteria, which determines that this formulation has a bactericidal action of broad-spectrum, offering potential antimicrobial activity [45].

Our results show that AgNPs synthesized from AgNO_3_ require concentration around micrograms for bacterial growth inhibition. The results of the present study were similar to those reported by Kanmani et al. [46], which gelatin/AgNPs nanocomposite films (30 and 40 mg) showed potent antimicrobial activity against Gram-positive and Gram-negative foodborne pathogens. AgNPs are capable of interacting physically with the cell surface of several bacteria. It is particularly important in the case of Gram-negative bacteria, where numerous studies report the adhesion and accumulation of AgNPs on the bacterial surface. Many studies have reported that AgNPs may impair cell membranes leading to structural changes, which make bacteria more permeable [47,48].

Mekkawy et al. [49] developed AgNPs stabilized with polymer, for which the MIC values were in the range of 0.93–7.5 and 3.75–15 µg/mL, respectively, Gram-positive (*Staphylococcus aureus*) and Gram-negative bacteria (*Escherichia coli*). Similar studies were also done by Ashmore et al. [50] showing that AgNP inhibited the growth of *E. coli* only at 0.621 mg/mL; and by Rath et al. [51] who obtained MIC of AgNPs against *S. aureus* and *P. aeruginosa* 5.8 ± 0.3 mg/ml and 7.4 ± 0.2 mg/mL, respectively.

**Figure 6:**
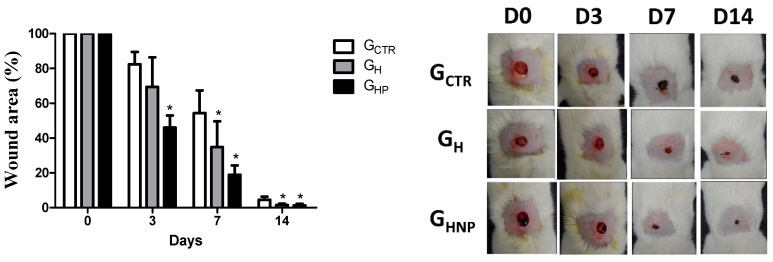
Non-splinted model showing the percentage of the non-epithelialized surface of the wound of the groups: G_CTR_ (Control Group), G_H_ (Group with hydrogel sodium alginate/gelatin (80:20), and G_HP_ (Group hydrogel with AgNP 4 mM AgNO_3_). All values are mean ± S.E. Statistical analysis comprised ANOVA followed by Tukey’s test. * *P* < 0.05 in relation to G_CTR_, G_H_, and G_HP_ groups, respectively (*n* = 21/group).

The wound healing capacity of the developed hydrogels was studied over 14 days, and the results are shown in Figure 6. The area of the wound has gradually decreased over time. Since no splinted wound model has been used to prevent contraction nor induce healing by re-epithelization, the healing shown in Figure 6 has been mainly by contraction mediated by myofibroblasts. As seen in the chart, the wound areas progressively decreased in both treated groups (G_HP_ and G_H_) significantly. The wound size was found to be reduced considerably in G_HP_, and G_H_-coated wounds on days 3, 7, and 14 of the postoperative period, compared to uncoated injuries (G_CTR_, p < 0.05, respectively). The effective action of the hydrogel with the incorporation of AgNPs in the group corresponding to G_HP_ has been observed, specifically on the third and seventh postoperative days. On the third day, the area of the wound was reduced by 46.03% when compared to the G_CTR_ groups, which decreased by 17.61% and G_H_ with a reduction of the wound area by 30.63%. On the seventh day, the G_HP_ was also effective in reducing the total area of the wound by 81.14%, while the G_H_ decreased by 65.11% higher than the G_CTR_ with 45.66% reduction of the injured area 45.66%. On the fourteenth day, the effective action of the G_HP_ and G_CTR_ hydrogels are possibly associated with the formation of the granulation tissue, characteristic of the last cicatricial phase, and because it is no longer corresponding to the inflammatory period, which is more sensitive to the action of microorganisms. It corroborates with the resolution of the use of AgNPs incorporated in the hydrogel, their antimicrobial activity made possible the reduction in the area of the wound mainly on the third and seventh day (Figure 6, left).

**Figure 7:**
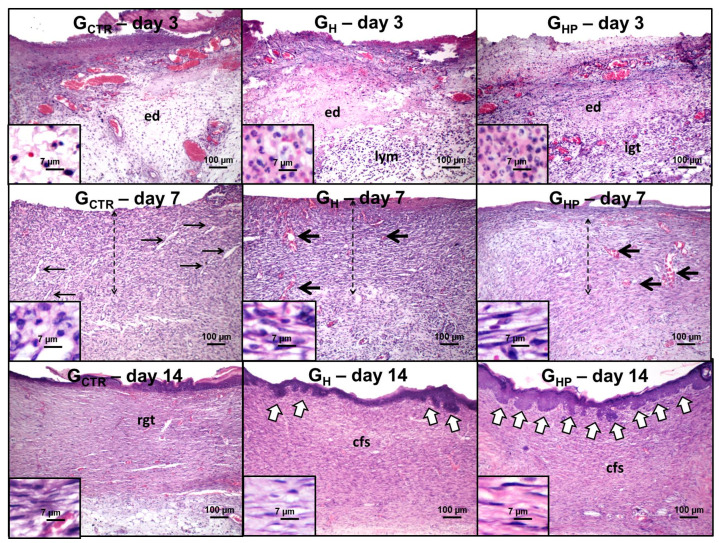
Photomicrographs of hematoxylin/eosin-stained histological sections representative of histological wound healing versus the time course of the experiment. Day 3: Wounds present intense edema (ed) and infiltration of polymorphonuclear neutrophils; note the lymphocyte-rich infiltrate (lym) and immature granulation tissue (igt) in the bottom of G_H_ and G_HP_, respectively (100×). Polymorphonuclear neutrophil (small lobular nuclei) and lymphocytes (dark round nuclei) are highlighted in higher magnification (800×). Day 7: Thick strips of granulation tissue are observed in all groups (dashed arrows); irregular and slit-shaped capillary blood vessels concentrated in the edge are seen in G_CTR_ (thin arrows), whereas dilated hyperemic vessels (thick arrows) are observed throughout the wound areas in G_H_ and G_HP_ (100×). Note the lower content of inflammatory cells in G_HP_. Stromal spindle cells (fibroblast and endothelial-like cells) are highlighted at higher magnification (800×). Day 14: Residual vascular granulation tissue (right) is observed in G_CTR_, but a cellular primary fibrous scar (cfb) is seen in G_H_ and G_HP_. Epithelial buddings (compatible with rudimentary cutaneous appendages) (white arrows) are found in the edges of the wound area in G_H_ but over the full epithelial surface in G_HP_ (100×). Stromal spindle cells (fibroblast-like cells) are highlighted in higher magnification (800×). G_CTR_ (Control Group), G_H_ (Group with hydrogel sodium alginate/gelatin (80:20), and G_HP_ (Group hydrogel with AgNP 4 mM AgNO_3_).

The histological wound healing occurred without intercurrence over the time-course of the experiment in all the studied groups (Figure 7). On day 3, an intense inflammatory infiltrate composed by polymorphonuclear neutrophils (PMN), and macrophages were observed throughout the wounded area of all groups. Interstitial edema was remarkable, particularly in G_CTR and_ G_H_. Interestingly, there was a more conspicuous chronic lymphocytic infiltrate in the bottom of G_H_ wounds, whereas G_HP_ presented the formation of immature granulation tissue, rich in hyperemic capillary vessels and proliferative endothelial-like spindle cells, in the bottom of the wounds. 

On day 7, granulation tissue containing plump, active fibroblasts forms was observed in all groups but at different maturation grades. The inflammation remained intense in G_CTR_, but there was a balance in the content of PMN, macrophages, and lymphocytes, which characterized a “persistent” subacute inflammatory infiltrate. Narrowed (slit-shaped) capillary vessels were concentrated on the edges and bottom of the wounds. In G_H_, the inflammatory response was essentially lymphocytic (chronic inflammation). The stromal spindle cells, interpreted as fibroblasts and endothelial cells, were densely dispersed throughout the wound area and arranged parallel to the wound surface. Most vessels were widely dilated and hyperemic. In G_HP_, the granulation tissue had more mature morphological features, such as reduction of the inflammatory response, higher content of hyperemic blood vessels, and more intense proliferation of fibroblast-like spindle cells. 

On day 14, there was the persistence of vascular granulation tissue in G_CTR_, whereas G_H_ and G_HP_ presented a cellular fibrous scar. All groups exhibited full epithelization, but only G_H_ and G_HP_ showed epithelial buddings compatible with the neoformation of rudimentary cutaneous appendages. Also, such buddings were limited to the scarred edges in G_H_ but scattered over the wound surface in G_HP_.

The pathological findings observed in G_CTR_ over the time-course of the experiment, expressed by acute inflammation, granulation tissue formation and primary scar development on day 3, 7 and 14, respectively, suggest that wound healing has occurred without intercurrences, which is also an indication that the control group can be used as an entirely acceptable parameter of normality of the pathophysiological steps of wound healing. 

The use of hydrogels containing AgNPs promoted histological changes in the healing tissue over the time course of wound healing, such as earlier development and maturation of granulation tissue. Synthetic products impregnated with silver used as dressings for the treatment of wounds have been demonstrated to act as a mechanical barrier against exogenous microorganisms, preserving the local temperature and maintaining the humidity of the wound environment [52]. Therefore, the application of sodium alginate/gelatin hydrogels containing AgNPs on the surface of the wounds may work as a biomechanical barrier, protecting the ulcerated bed from the microbial contamination. The silver-induced antibacterial effect has been associated with its ability to interact with bacterial plasma membranes, proteins, and enzymes involved in vital cellular processes, such as the electron transport chain [53]. AgNPs incorporated into dressings have significantly decreased wound-healing time likely as a result of increased bacterial clearance from infected wounds [54,55].

However, other silver nanoparticles-derived biological properties might have played a role in the acceleration of granulation tissue formation and maturation on the initial stages of wound healing. AgNPs have been demonstrated to reduce the production of the inflammatory cytokines, such as nitric oxide and prostaglandin E2, in lipopolysaccharide-induced RAW264.7 cells [56]. Hence, the silver nanoparticles-induced reduction of the inflammatory cytokines release might have also played a role in the improvement of granulation tissue formation. 

The dermal connective tissue of the G_HP_ group showed earlier formation of mature hypovascular primary scars, but no pathological signs of hypercollagenization was observed. It has been previously reported that silver nanoparticles can reduce the levels of transforming growth factor β (TGF-β) expression while increasing interferon (IFN)-γ levels until full wound closure [57]. IFN-γ has been demonstrated to inhibit fibroblast proliferation and matrix production [58] and induce myofibroblasts apoptosis [59], whereas enhanced expression of TGF-β1 mRNA was found in both keloids and hypertrophic scars [60]. Hence, the modulation of TGF-β/ IFN-γ production may play a role in the positive effects of silver on wound healing.

At the final stages of wound healing, healed skin treated with sodium alginate/gelatin hydrogels containing AgNPs presented a well-stratified epidermis, complete with basal, spinous, granular, and cornified layers, with epithelial buddings interpreted as rudimentary cutaneous appendages. Also, collagen tissue was well-formed, with long thick gross collagen fibers parallel-arranged. These results suggest that the silver-containing hydrogels improved both dermal and epidermal re-establishment. The precise mechanisms underlying such biological effects are not fully clarified yet. It has been demonstrated that silver nanoparticles can induce contraction of the wounds in mice as a result of increased keratinocyte migration and proliferation [61]. However, further studies are needed to find out whether the formation of rudimentary cutaneous appendages in the G_HP_ group, but not in the others, result from AgNPs-induced stimulation of keratinocyte proliferation and differentiation or would be an indirect response secondary to the improvement of the early phases of wound healing.

Our study suggests that hydrogels containing AgNPs provide significant benefits on wound healing in a rodent model; herein, we provide the evidence that AgNPs can accelerate granulation tissue formation and maturation and earlier development of the primary collagen scar and rudimentary cutaneous appendages. 

## 4. Conclusions

This study aimed to produce a safe, biocompatible formulation based on AgNPs for wound healing, for easy application and of low environmental impact. No inorganic solvents have been used nor any complex methodology that needs high energy. The use of natural polymers as sodium alginate and gelatin is a cost-effective approach for the production of a biocompatible hydrogel that can be easily loaded with antimicrobial AgNPs with healing properties. Compared to synthetic ones, natural polymers are of lower cost, non-toxic, less abrasive, and are environmentally friendly. The developed hydrogels demonstrated to be non-cytotoxic against fibroblasts, and their antimicrobial activity was confirmed in vitro using Gram-positive (*Staphylococcus aureus*) and Gram-negative (*Pseudomonas aeruginosa*) bacteria. The wound healing capacity of AgNPs hydrogels was studied over 14 days in Wistar rats highlighting that AgNPs can accelerate tissue formation and promote earlier development of primary collagen scars.

## Figures and Tables

**Table 1:** MIC of the gelatin, sodium alginate, and silver nitrate solutions, hydrogels with silver nanoparticles at different concentrations (1 mM, 2 mM, and 4 mM).

Bacteria	Gelatin	Sodium Alginate	AgNO_3_	Hydrogel 1 mM	Hydrogel 2 mM	Hydrogel 4 mM
Gram-negative *Pseudomonas aeruginosa* ATCC 27853	Bacterial Growth	Bacterial Growth	No bacterial growth	1.050 µg.mL^−1^	1.050 µg.mL^−1^	00.50 µg.mL^−1^
Gram-positive *Staphylococcus aureus* ATCC 25923	Bacterial Growth	Bacterial Growth	No bacterial growth	130 µg.mL^−1^	130 µg.mL^−1^	53.0 µg.mL^−1^
