# Correction: Diniz et al. Silver Nanoparticles-Composing Alginate/Gelatine Hydrogel Improves Wound Healing In Vivo. *Nanomaterials* 2020, *10*, 390

**DOI:** 10.3390/nano12224071

**Published:** 2022-11-18

**Authors:** Flavia Resende Diniz, Romerito Cesar A. P. Maia, Lucas Rannier M. de Andrade, Luciana Nalone Andrade, Marco Vinicius Chaud, Classius Ferreira da Silva, Cristiane Bani Corrêa, Ricardo Luiz C. de Albuquerque Junior, Luiz Pereira da Costa, Su Ryon Shin, Shabir Hassan, Elena Sanchez-Lopez, Eliana Barbosa Souto, Patricia Severino

**Affiliations:** 1 Tiradentes University (UNIT) and Institute of Technology and Research (ITP), Av. Murilo Dantas 300, Aracaju 49032-490, Brazil; 2 Department of Pharmaceutical Technology, Faculty of Pharmacy, University of Coimbra, Pólo das Ciências da Saúde, Azinhaga de Santa Comba, 3000-548 Coimbra, Portugal; 3 Department of Technological and Environmental Processes, Sorocaba University (UNISO), Rod. Raposo Tavares, Km 92.5, Sorocaba 18023-000, Brazil; 4 Department of Chemical Engineering, Federal University of São Paulo (UNIFESP), Rua Prof. Artur Riedel, 275, Diadema 09972-270, Brazil; 5 Department of Morphology, Federal University of Sergipe (UFS), Avenida Marechal Rondon, São Cristovão 49100-000, Brazil; 6 Center for Biomedical Engineering, Department of Medicine, Brigham and Women’s Hospital, Harvard Medical School, 65 Landsdowne Street, Cambridge, MA 02139, USA; 7 Department of Pharmacy, Pharmaceutical Technology and Physical Chemistry, Faculty of Pharmacy and Food Sciences, Av. Joan XXIII 27-31, 08028 Barcelona, Spain; 8 Institute of Nanoscience and Nanotechnology (IN2UB), University of Barcelona, Av. Joan XXIII 27-31, 08028 Barcelona, Spain; 9 Biomedical Research Networking Centre in Neurodegenerative Diseases (CIBERNED), 28031 Madrid, Spain; 10 CEB—Centre of Biological Engineering, University of Minho, Campus de Gualtar, 4710-057 Braga, Portugal; 11 Tiradentes Institute, 150 Mt Vernon St, Dorchester, MA 02125, USA

## Error in Figure

In the original publication, there was a mistake in Figure 6 as published [1]. Upon the building up of the combined image of day 3, the authors misplaced the panel corresponding to G_H_ while the panel corresponding to G_CTR_ was copied twice.


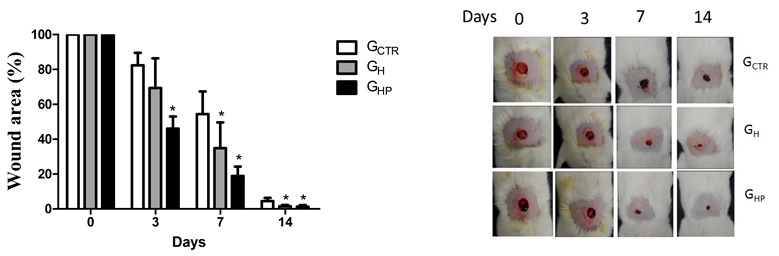


The authors do apologize for this mistake. The revised version corroborates the conclusions stated in the first version of the article; the wound size was reduced considerably over the 14 days of the postoperative period, compared to the uncoated injuries. The corrected Figure 6 appears below.

**Affiliation Correction**

In the published publication, there was an error regarding the affiliations for **Classius Ferreira da Silva** and **Elena Sanchez-Lopez**. The original affiliations **4,7,8** should be updated as follows.

Affiliation 4: “Department of Exact Sciences and Earth, Federal University of São Paulo (UNIFESP), Rua Prof. Artur Riedel, 275, Diadema CEP 09972-270, Brazil” updated to “Department of Chemical Engineering, Federal University of São Paulo (UNIFESP), Rua Prof. Artur Riedel, 275, Diadema 09972-270, Brazil”.

Affiliations 7 and 8: “7 Department of Pharmacy, Pharmaceutical Technology and Physical Chemistry, Faculty of Pharmacy and Food Sciences and Institute of Nanoscience and nanotechnology (IN2UB), University of Barcelona, Av. Joan XXIII 27-31, 08028 Barcelona, Spain”, “8 CIBERNED Centros de Biomedicina en Red de Enfermedades Neurodegenerativas, Facultat de Farmàcia, Universitat de Barcelona, 08028 Barcelona, Spain” should be update to “7 Department of Pharmacy, Pharmaceutical Technology and Physical Chemistry, Faculty of Pharmacy and Food Sciences, Av. Joan XXIII 27-31, 08028 Barcelona, Spain”, “8 Institute of Nanoscience and Nanotechnology (IN2UB), University of Barcelona, Av. Joan XXIII 27-31, 08028 Barcelona, Spain”, “9 Biomedical Research Networking Centre in Neurodegenerative Diseases (CIBERNED), 28031 Madrid, Spain”.

**Author Name Correction**

The author’s name “Lucas Rannier Andrade” should be changed to “Lucas Rannier M. de Andrade”.

The authors apologize for any inconvenience caused and confirm that the scientific conclusions are unaffected. This correction was approved by the Academic Editor. The original publication has also been updated.

## Figures and Tables

**Figure 6:**
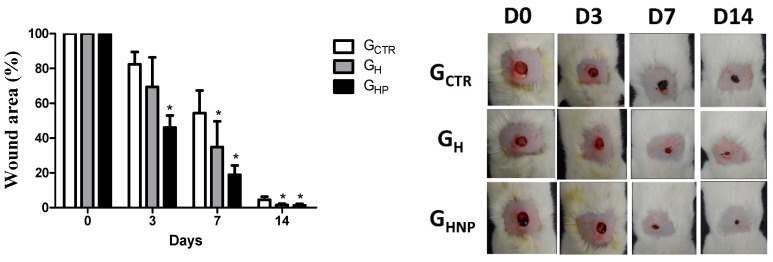
Non-splinted model showing the percentage of non-epithelialized surface of the wound of the groups: G_CTR_ (control group), G_H_ (group with hydrogel sodium alginate/gelatin (80:20), and G_HP_ (group with hydrogel with AgNP 4 mM AgNO_3_). All values are mean ± S.E. Statistical analysis by ANOVA followed by Tukey’s test. * *p* < 0.05 in relation to G_CTR_, G_H_, and G_HP_ groups, respectively (*n* = 21/group).
